# Systemic Reflections on Knowledge Transfer: Comment on "Sustaining Knowledge Translation Practices: A Critical Interpretive Synthesis"

**DOI:** 10.34172/ijhpm.2022.7559

**Published:** 2022-09-21

**Authors:** Joachim P. Sturmberg

**Affiliations:** ^1^College of Health, Medicine and Wellbeing, University of Newcastle, Callaghan, NSW, Australia.; ^2^International Society for Systems and Complexity Sciences for Health (ISSCSH), Waitsfield, VT, USA.

**Keywords:** Knowledge Transfer, Knowledge Translation, Systems Thinking, Complex Adaptive Organisations, Organisational Learning, Philosophy of Science

## Abstract

The systemic failure of organisational learning should not come as a surprise – after all every system delivers exactly what it is designed for. Knowledge management/transfer is a property of the organisational system rather than a particular technique. Hence, knowledge management/transfer is about the contextual framing in which learning focused on understanding can occur. Looking through a system lens any research field can be defined as a complex adaptive organisation, and its culture determines if and how learning and knowledge transfer (or shared learning) can occur. Creating and maintain a learning culture requires leadership that perpetuates continuous dialogues to achieve tacit and explicit knowledge exchange.

*We’ll get to the truth quicker if we don’t worry about logic. *

**Louise Erdrich - ** American author

 “*The health policy and systems research literature increasingly observes that knowledge translation (KT) practices are difficult to sustain. An important issue is that it remains unclear what sustainability of KT practices means and how it can be improved*”^1^ Borst, Wehrens and Bal’s, based on an extensive literature search and thematic synthesis, describe the systemic *failures *(or at a minimum difficulties)* of organisational learning*. That should not come as a surprise as every system always delivers exactly what it is designed to deliver.^2^ Put differently, failure of KT is an inherent property of the system (the organisation people work at) that fails to achieve KT (and thus not a problem of the people themselves).^3^

 The core argument of this short commentary is that knowledge management/transfer, rather than being a *technique *(in Ackoff’s terms instructions^4^), in the first instance is one of *contextual framing* and thus the creation of *understanding.*^4^

## Knowledge Transfer = *Systemic Organisational Learning*

 Borst et al also tacitly acknowledges that health policy and systems research is a heterogeneous field that lacks a clear definition of purpose. Any research field reflects an organisational frame, and thus can be defined – looking through a systems lens – as a *complex adaptive organisation*. It is the organisational context that provides the environment in which learning and knowledge transfer (or shared learning) occurs. Learning, broadly speaking, is the acquisition of new understandings, knowledge, behaviours, skills, values and attitudes.

 Not all organisations foster shared learning, and some indeed prevent it from occurring based on a command-and-control philosophy where information and knowledge is only shared on an at-needs basis. Such organisation have a bias towards success (paying lip-service to learning from failure) and action (resulting in exhaustion and lack of time to reflect), and tacitly demand people to fit in (diminishing creativity).^5^

 In contrast, *complex adaptive organisations* have a set of key characteristics that explain the *why, what* and *how* of the organisation’s approaches to achieve its tasks. Such organisation:

1. *Understand their purpose/focus* – WHY are we here? WHAT do we want to achieve?
2. *Define specific goals to achieve* – WHAT exactly do we want to deliver within a given time frame?
3. *Understand their core values* – WHAT are the values that do not change even if our circumstances change? They must be consistent with the purpose of the organisation; and
4. *Articulate their ‘core operational rules’ (aka “simple rules”)* – How do we interact? What are the key ways (or what are – typically – the 3-5 principles) that define ‘how we do business’ in this organisation?^6^

By implication, *complex adaptive organisations* are learning organisations – they have a collaborative purpose-focused approach, facilitate a continuous dialogue between tacit and explicit knowledge exchanges,^7^ and embrace a collective and collaborative problem-solving approach. The free flow of information and knowledge amongst all its members regardless of their role within the organisation is valued as the *sine-qua-non* for success, and constantly reinforced and upheld by its leadership (for more detail see Heifetz^8,9^) (Box 1).

**Box 1. Perspectives on Organisational Learning and Learning Organisations**

** Definitions of Organisational Learning/Learning Organisation**

Organizational learning is a process of detecting and correcting error.^10^

Organizational learning means the process of improving actions through better knowledge and understanding.^11^

Organizational learning occurs through shared insights, knowledge, and mental models…[and] builds on past knowledge and experience—that is, on memory.^12^

Learning organizations [are] organizations where people continually expand their capacity to create the results they truly desire, where new and expansive patterns of thinking are nurtured, where collective aspiration is set free, and where people are continually learning to see the whole together.^13^

Learning organizations are characterized by total employee involvement in a process of collaboratively conducted, collectively accountable change directed towards shared values or principles.^14^

The Learning Company is a vision of what might be possible. It is not brought about simply by training individuals; it can only happen as a result of learning at the whole organization level. A Learning Company is an organization that facilitates the learning of all its members and continuously transforms itself.^15^

## Knowledge Transfer – “Work” or “*Absorption/Integration*”

 “*This ‘sustaining work’ is an interplay of three processes: ( i ) translating, (ii) contexting, and (iii) institutionalising. Translating refers to activities aimed at constructing and extending networks. Contexting emphasises the activities needed to create contexts that support KT practices. Institutionalising addresses how actors create, maintain, and disrupt institutions with the aim of sustaining KT practices.”*^1^ Borst, Wehrens and Bal’s observation of knowledge transfer as ‘work’ describes why most organisations are ‘non-learning organisations.’^5^ They are dysfunctional organisations whose key flaw is the failure to define its purpose. It should be unsurprising that it becomes necessary to ‘enforce’ processes for knowledge transfer in the hope that these will achieve the desired outcomes – they may, but more like they do not, and not in a sustainable way.

 The purpose-focused and purpose-driven approach of *complex adaptive organisations* fosters constant translation of observations and insights – learning (or the creation of understanding) is emergent and occurs by absorption and integration within the well-known context of the organisation. Knowledge transfer, new knowledge creation, and KT^16^ all enhance understanding^4^ and are a defining characteristic of the *culture* of these organisations.^7,17^

## The Way Forward – Embrace *Complex Adaptive Systems* Approaches

 Rather than needing more studies we need more pragmatism. Logically understanding the difficulties of achieving and sustaining knowledge transfer is unlikely to achieve our desired goal. We already know that the systemic – and by implication – cultural characteristics of an organisation determine failure and success.^17^ We need leadership that can facilitate the necessary organisational change required to create an environment for continuous dialogues to achieve tacit and explicit knowledge exchanges that ultimately result in new knowledge creation (Figure).^7,8,17^ The Cynefin *framework* offers a useful frame to achieve greater understandings^4^ and to meaningfully transfer of these understandings across organisations.^18^

**Figure:**
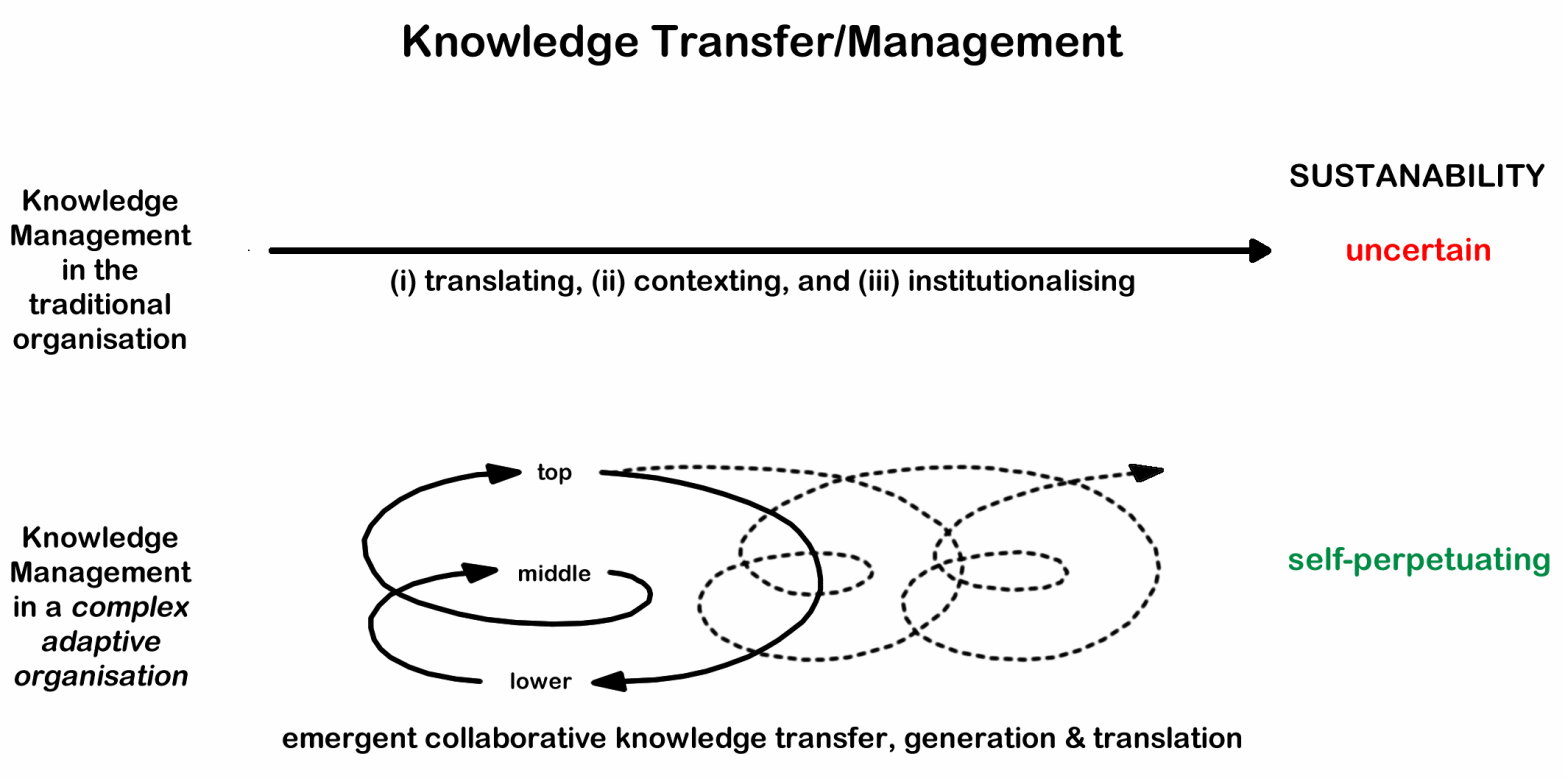
Differences in Knowledge Management.

## Ethical issues

 Not applicable.

## Competing interests

 Author declares that he has no competing interests.

## Author’s contribution

 JPS is the single author of the paper.
